# Exploring the Creative Process: Integrating Psychometric and Eye-Tracking Approaches

**DOI:** 10.3389/fpsyg.2018.01931

**Published:** 2018-10-09

**Authors:** Dorota M. Jankowska, Marta Czerwonka, Izabela Lebuda, Maciej Karwowski

**Affiliations:** ^1^Department of Educational Sciences, The Maria Grzegorzewska University, Warsaw, Poland; ^2^Institute of Psychology, University of Wrocław, Wrocław, Poland

**Keywords:** psychometrics, creative process, Test of Creative Thinking-Drawing Production, eye-tracking, thinking aloud

## Abstract

This exploratory study aims at integrating the psychometric approach to studying creativity with an eye-tracking methodology and thinking-aloud protocols to potentially untangle the nuances of the creative process. Wearing eye-tracking glasses, one hundred adults solved a drawing creativity test – The Test of Creative Thinking-Drawing Production (TCT-DP) – and provided spontaneous comments during this process. Indices of visual activity collected during the eye-tracking phase explained a substantial amount of variance in psychometric scores obtained in the test. More importantly, however, clear signs of methodological synergy were observed when all three sources (psychometrics, eye-tracking, and coded thinking-aloud statements) were integrated. The findings illustrate benefits of using a blended methodology for a more insightful analysis of creative processes, including creative learning and creative problem-solving.

## Introduction

While scholars generally agree that creativity leads to ideas and products that are novel (original) and meaningful (useful, relevant) (see Runco and Jaeger, 2012), much less agreement is observed when it comes to the creative process. For good reasons, though: dynamism of the process and variety of mechanisms involved in generation and explorations of ideas make it challenging to capture.

Diverse conceptualizations of how, when, and why people create have resulted in a set of quite isolated measurement approaches that, taken together, make effective synthesis of previous findings difficult. Are there any common findings that may be considered as regularities of the creative process, despite the methods applied? Or perhaps different measurement approaches, by definition, can capture only some aspects of the creative process?

In this article, we analyze different perspectives and include a variety of methods by integrating the more traditional psychometric approach (usually based on scores obtained in creative thinking tests) with an analysis of metacognitive and self-regulation mechanisms engaged within the process (measured by think-aloud protocols), and the parameters that reveal the way attention functions during this process (measured by the eye-tracking methodology). We posit that such triangulation holds the promise to result in a more complex and comprehensive look at the process itself. The approaches we use in the study described below not only give us an opportunity to measure real-time (Schwarz, 2012) dynamics of the process, but also potentially catch the interaction between the person (or actor; see Glăveanu, 2013) and the outcome (or product; see Botella et al., 2013), and to include metacognitive aspects of the process into our analyses.

There are different theoretical and methodological views on the nature of the creative process and its measurement. Below, we briefly focus on three of them, which we consider most relevant from the perspective of our investigation. The first, most classic perspective divides the creative process into a number of different, sequential or recursive stages, or phases (e.g., Wallas, 1926). More contemporary extensions describe the process in terms of the most important mental operations and behaviors within each of the stages (Isaksen et al., 2000; Sawyer, 2012). The role of cognitive processes (e.g., Mumford et al., 1991, 1997) or different facets (Amabile, 1996) during the process of creating or problem solving is analyzed as well. The classic stage models often utilize a wide range of methods, starting from qualitative interviews (Perry, 1999) or observations (Csikszentmihalyi, 1996; Dunbar, 1997), all the way to historical case studies of eminent creators (Weisberg, 1986) or computational models based on the archival study of individual creative episodes taken from the notebooks of scientists (e.g., Langley et al., 1987; Kulkarni and Simon, 1988).

The second view on the creative process, i.e., the creative cognition approach (Finke et al., 1992), emphasizes and micro-analytically investigates the cognitive mechanisms as the core of the creative thought. For example, the geneplore model (Finke et al., 1992) specifies the creative process as a set of basic cognitive processes that increase the likelihood of a creative output. The nature of both generative and exploratory processes – two main phases in the geneplore model – can be described and potentially modeled thanks to the understanding of detailed operations and processes engaged in both these phases. The creative cognition approach largely benefited from a convergence strategy (Ward, 2001): anecdotal facts about great creative discoveries served as an inspiration that allowed to hypothesize specific phases and mental operations involved in creative thinking that were subsequently rigorously examined in controlled laboratory experiments. Studies inspired by the creative cognition approach usually focus on intensive, experimental laboratory task – the so-called creative generation tasks. Participants are presented with open-ended problems (e.g., drawing animals that may exist on other planets) and their solutions are scored in terms of creativity or originality. Importantly, the creative cognition approach is focused on both the outcome and the process itself.

The third approach refers to psychometrics that has for decades been the predominant approach to understanding individual differences in creativity and, to a lesser extent, the creative process. Not surprisingly, psychometricians tended to rely on divergent thinking tests or other standardized methods, such as scales or questionnaires (see Hocevar, 1981). Although the expansion of purely psychometric work has been criticized as providing, at best, a fragmented and incomplete (see e.g., Baer, 1994; Glăveanu, 2014) and, at worst, invalid picture of creativity, usefulness of this approach goes without saying (see Plucker and Runco, 1998 for a discussion). Psychometric works identified several important effects in the psychology of creativity, such as the serial-order effect (Beaty and Silvia, 2012), the threshold hypothesis (Jauk et al., 2013; Karwowski et al., 2016a), or the fourth-grade slump in creativity (Krampen, 2012). What’s more, current works that utilize psychometric tasks tend not only to explore the overall creativity of the outcomes created (e.g., responses in the unusual uses tasks), but also the dynamics of the process itself (Gilhooly et al., 2007; Beaty and Silvia, 2012; Silvia et al., 2017).

### Exploring the Dynamics of the Creative Process

The creative process has been examined with the use of both qualitative and quantitative methods that permit differentiating the stages of the creative process, e.g., among professional artists (Botella et al., 2013) or screenplay writers (Bourgeois-Bougrine et al., 2014). Moreover, contemporary studies have demonstrated an analysis of temporal dynamics in creative ideation (Gilhooly et al., 2007; Beaty and Silvia, 2012; Silvia et al., 2017) – not necessarily restricted to individuals, but also in dyads (Glăveanu et al., 2018). They also applied visual-verbal protocols to explore the meshed modes of creative thinking in time (Pringle and Sowden, 2017). Likewise, novel techniques and measures that allow for better understanding of the creative process are being developed. Conceptual Clock-face in testing the role of distance in conceptual processing (Hocking and Vernon, 2017) and Mode Shifting Index to assess shifts between associative and analytic modes of creative thought (Pringle and Sowden, 2016) are but two examples. In the same vein, data analysis methods are becoming more sophisticated – researchers not only combine qualitative and quantitative strategies (Bourgeois-Bougrine et al., 2014), but they also routinely use multivariate analyses (with semantic component analysis; Botella et al., 2013) and multilevel modeling (Glăveanu et al., 2018). The natural advantage of more synergistic approaches lies in the potential to combine a wider set of data, allowing for a more complex understanding of the creative process.

Thus, we observe a growing number of studies that focus on developing or adapting methods, and it seems that creativity scholars strive to combine different perspectives and use more blended approach. Does it mean that psychometric measures of creative abilities and creative process will eventually be discarded? We doubt it. Instead, we suggest putting more effort into *dynamizing creativity tests* that would allow for a more fruitful analysis of the process rather than the output alone. Such a blended, multi-method approach was effectively used to analyze the dynamic nature of the creative learning process (e.g., Gajda et al., 2017). Gajda et al. (2017) combined the qualitative (observations, audio-recorded interactions) and quantitative (measures of creativity and academic achievement) method to explore interpersonal characteristics of creativity in a classroom. Here, we take a more intrapersonal orientation by exploring the role of strategies and self-regulation during a creativity test.

### How Do Methodological Innovations Inform Our Understanding of the Process?

Previous findings showed that individuals implement various strategies – from the more structured (Sawyer, 2012) to more isolated (Root-Bernstein and Root-Bernstein, 2001) – during the creative process. Other classifications defined strategies as experiential – derived from episodic personal memory – or semantic, thus based on abstract, conceptual knowledge (Walker and Kintsch, 1985; Vallee-Tourangeau et al., 1998). Gilhooly et al. (2007) classified the strategies obtained using think-aloud protocols into Memory, Property, Broad Use and Disassembly strategies – and demonstrated that different strategies operate at the initial and later creative process stages.

Analysis of strategies involved in the performance phase (e.g., critical thinking, ideation, imagery) seems to be specifically relevant to their relationship with self-regulated learning (Rubenstein et al., 2017). According to this approach, creative process strategies may support learning strategies, because of their relevance for self-regulation. Effective self-regulation processes are crucial for successful transformation of creative ideas into creative products (Ivcevic and Nusbaum, 2017). Two broad groups of self-regulation processes in creativity have been identified in previous studies: [1] revising and re-strategizing, and [2] sustaining and maintaining effort. The first set involves continual exploration and revision (Csikszentmihalyi and Getzels, 1971; Csikszentmihalyi, 1988). The second set involves both planning and implementation operations. While appreciating this broad categorization, we posit that even a more detailed analysis of strategies may be necessary to describe different idiosyncrasies of the creative process. On the broad level, it seems that strategies focused on generation and exploration may be important during the initial phases of the creative process, while those related to monitoring and control activities are engaged more steadily across the entire process – meaning not only generation of initial ideas, but also their combinations and polishing. In the literature, theoretical premises and empirical evidence demonstrate the impact of metacognitive strategies on creativity (Pesut, 1990) and creative problem solving (Hargrove and Nietfeld, 2015); thus, we assume a non-trivial role of metacognitive strategies and mechanisms that refer to affective-evaluative activity. It is also widely accepted that different creative self-beliefs are engaged in the creative process and their role is vital in initiating the activity, but especially in expending effort in the creative process (see e.g., Karwowski and Beghetto, 2018). Therefore, we expect that self-efficacy or affect-based evaluative behaviors may be prominent during the final stages of the creative process as well.

Another rapidly developing line of a creative process analysis applies neuroscience and behavioral methods (e.g., Beaty et al., 2016, 2018). Although a detailed overview of these approaches is outside of the scope of this article (see Benedek, 2018 for a review), these studies provide compelling evidence of the integrative character of the creative process. It has been demonstrated that the creative process integrates brain default and executive networks, thus providing the evidence that mind-wandering as well as controlled thinking are simultaneously engaged in the creative process, and – importantly – free as well as controlled processes play roles during all phases of the process. In a similar vein, eye-tracking methods are applied to follow the attention mechanisms involved in the process (e.g., Vartanian, 2009; Beaty et al., 2014; Agnoli et al., 2015; Benedek, 2018). Researchers linked types of processing within the creative process with focused or defocused (Howard-Jones, 2002; Gabora and Ranjan, 2013), or internal versus externally directed attention (Benedek, 2018). Indeed, data obtained thanks to the eye-movement tracking methodology are quite informative for understanding the shifts and subprocesses during idea generation and evaluation (e.g., Ueda et al., 2015). Recent studies demonstrate that the idea generation phase is accompanied by reduced micro-saccade activity and by longer and more frequent blinks (Benedek et al., 2017; Walcher et al., 2017). Findings also suggest that solving insight problems goes with more extended blinks and more gaze aversion (Salvi et al., 2015). An occulometric measure (specifically eye-blink rate; EBR) is an attentional marker of mind-wandering during creative thinking (e.g., Baird et al., 2012; Hao et al., 2015).

### The Present Study

The main goal of this exploratory study was to attempt to integrate a relatively static, psychometric approach with a more dynamic, process-based analysis of attention and metacognition functioning during the creative process. To this end, we explored how participants solved the figural creativity test [Test of Creative Thinking-Drawing Production (TCT-DP)], but instead of focusing solely on its outcome (or the final score), we used eye-tracking methodology and thinking-aloud protocols with the hope to provide a more nuanced and dynamic analysis of the process.

## Materials and Methods

### Participants

One hundred participants (50 female and 50 male) aged between 18 and 40 years (*M* = 28.82, *SD* = 7.33) participated in this study. Participants were recruited on the main streets in the center of Warsaw, the capital of Poland and invited to the lab. Participants were remunerated for their time with a one-time payment of 50 PLN (equivalent of approximately 12 euro).

### Measures

#### Test for Creative Thinking-Drawing Production (TCT-DP)

**FIGURE 1:**
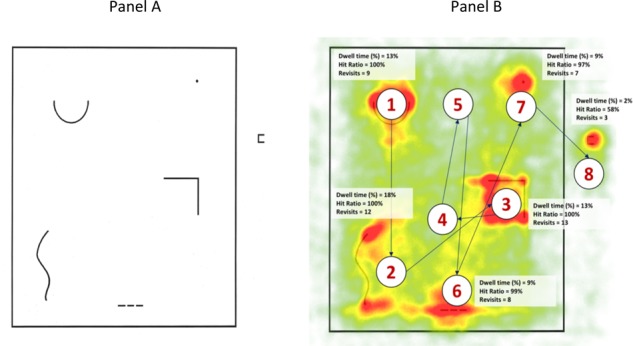
Test of Creative Thinking-Drawing Production (TCT-DP) testing sheet **(A)**, saccades and fixations while solving the TCT-DP **(B)** (numbers illustrate the most typical sequence of participants gazes).

We used Urban and Jellen (1996) TCT-DP. Participants were asked to complete a drawing with six elements placed asymmetrically on a test sheet (see **Figure 1**, panel A). Assessment of the TCT-DP includes fourteen detailed criteria: (1) continuations, (2) completions, (3) new elements, (4) connections made with a line, (5) connections that contribute to a theme, (6) boundary breaking that is fragment-dependent, (7) boundary breaking that is fragment-independent, (8) perspective, (9) humor and affectivity, (10) unconventionality: manipulation of the test material; (11) unconventionality: surrealistic or abstract elements; (12) unconventionality: use of symbols or signs; (13) unconventionality: unconventional usage of the given fragments and (14) speed. As this study was untimed, we relied on 13 instead of 14 criteria. Speed, as an optional, additional criterion of the test (Urban and Jellen, 1996), was omitted, because the methodology used (especially think-aloud protocols) made the process longer than usual. The final TCT-DP result is the sum of points obtained in all tested criteria. The total score in TCT-DP (without considering speed) my range between 0 and 66 points. Previous studies (e.g., Karwowski et al., 2016b) confirmed validity and reliability of the TCT-DP. In this study, the internal consistency of TCT-DP was comparable to previous studies (α = 0.74). The TCT-DP was scored independently by two coders (second and third author with an excellent reliability: *r* = 0.987).

#### Eye-Tracking

Participants solved the TCT-DP wearing eye-tracking glasses (SensoMotoric Instruments, SMI) with a temporal resolution of 120 Hz. We used the manufacturer’s software to calibrate and compute the eye movement parameters: fixations and saccades. Before the study, there was a 4-point calibration procedure. Six main indices were analyzed for each of the area of interests – main elements of the TCT-DP (AOI, see **Figure 1**), specifically: (1) entry time – time in milliseconds to the first fixation within the AOI, (2) dwell time – total time in milliseconds spent on all AOIs in total, (3) hit ratio – the number of participants who fixated within the AOI, (4) revisits – the number of revisits to the specific AOI, (5) average fixation –length of the average fixation within the AOI, and (6) length of the first fixation within the AOI.

#### Thinking-Aloud Protocols

Metacognitive and self-regulation mechanisms engaged in the creative process were measured by participants’ statements and activities during the process of completing the TCT-DP. The think-aloud statements were audio-recorded after securing proper consent from all participants.

### Procedure

The study was conducted individually and lasted between 20 and 45 min. After a short introduction of the goals of the study, obtaining informed consent, and calibrating the eye-tracking glasses, participants filled the TCT-DP. They were instructed to complete a drawing and think aloud during the process – this request was repeated by the researcher if participants tended to draw in silence. Additionally, the following points were emphasized: (1) participants should perform the test in a way that they would if they were not thinking aloud; (2) they should verbalize all thoughts that occur while solving this test, and (3) they should be natural about their reactions. Moreover, participants solved one additional test and filled one questionnaire outside of the scope of this study.

### Ethics Statement

This study was carried out after obtaining written informed consent from all subjects. All subjects were informed about the goals of the study and provided informed consent. The protocol was approved by the first author’s Institutional Review Board (decision number 128-2016/2017).

## Results

The results are presented in four steps. We start with a basic description that illustrates how the process of filling the TCT-DP looked. Then, we switch to the question of whether it is possible to predict psychometric results obtained in the TCT-DP based on eye-tracking results. The third step involves a more detailed analysis of metacognitive strategies and activities during the creative process among individuals who obtained the highest and lowest scores in the TCT-DP. The last step of analyses examined whether metacognitive strategies are related to visual activity during the creative process, as measured by eye-tracking indices.

### The Process of Completing the TCT-DP

The average total score obtained in the TCT-DP was in line with previous studies on similar samples in Poland (Gralewski et al., 2016; Karwowski et al., 2016b): *M* = 19.15, *SD* = 9.45. Thus, the results did not suggest that the use of eye-tracking glasses combined with retrospective think-aloud influenced the results due to verbal overshadowing or having the glasses *per se*.

As illustrated on **Figure 1** (panel B), almost all participants focused on five main elements of the test placed within the border, yet almost half of them (42%) completely ignored and omitted the small unfinished square outside. The most typical path of saccades included exploration of the element placed in the upper-left side of TCT-DP (the semicircle) as first, switching to the curve placed in the bottom-left corner next, and then exploring the center-right unfinished square (right angle), bottom dashed line, and the upper-right dot. On average, participants spent most time looking at the bottom curve shape (18% of the total dwell time), then the semicircle and the unfinished square (13% each), while less time was devoted to looking at the line and dot (both 9%), and the unfinished small square outside the frame (2%). Overall, almost 2/3 (63%) of all registered glances were assigned to the six elements of the tests – the remaining ones were linked to the places between elements. The number of revisits to main elements of interests ranged from 7 in the case of the dot to 13 in the case of the unfinished square.

### Visual Activity and TCT-DP Results

To examine the extent to which the basic indices registered during eye-tracking are able to predict the psychometric results obtained in the TCT-DP, we proceeded with a two-step procedure. First, we estimated Pearson’s correlations between the main indices obtained in the eye-tracking study and the total score of the TCT-DP. Second, we used hierarchical clustering to identify groups with different profiles of gaze distribution and compared TCT-DP scores across the groups. Given the exploratory character of our study and a large number of independent tests, in all cases, we used the Holm–Bonferroni sequential correction for multiple comparisons (Holm, 1979).

As illustrated in **Table 1**, the links between ET indices and TCT-DP scores were significant and robust in terms of the effect size. The more time the participants spent on looking at the main areas of interest, the higher their scores were. Similarly, the more fixations within the AOIs were recorded and revisits to AOIs were found, the higher the scores in the test were. A negative correlation was obtained between the percentage of the dwell time within a single AOI and the total score in TCT-DP. In other words, the more intensively and dynamically the participants explored the test sheet, the higher their scores were, while the more exclusive focus on the certain part of the test resulted in lower scores on average.

**Table 1:** Pearson’s correlations and 95% confidence intervals between the total score in the Test of Creative Thinking-Drawing Production (TCT-DP) and main indices obtained in ET study.

TCT-DP with:	Pearson’s *r*	Lower 95% CI	Upper 95% CI
Entry time (ms)	0.49***	0.33	0.63
Dwell time (ms)	0.41***	0.23	0.56
First fixation duration (ms)	–0.03	–0.23	0.17
Revisits	0.46***	0.30	0.61
Fixation count	0.44***	0.27	0.59
Dwell time within AOI (%)	–0.35***	–0.51	–0.16
Average fixation duration (ms)	–0.03	–0.23	0.17

*N = 100, ^∗∗∗^p < 0.001 – given several independent tests, the Holm–Bonferroni correction was used to control for Type-I error.*

**FIGURE 2:**
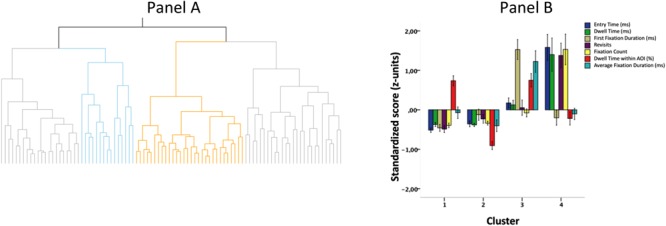
Dendrogram illustrating the number of clusters **(A)** and cluster profiles **(B)**. Error bars on **B** denote standard errors of the mean.

Hierarchical cluster analysis (Revelle, 1979; Yim and Ramdeen, 2015) on standardized scores of all ET indices performed with the use of Ward agglomeration technique suggested a four-cluster solution (see **Figure 2**, panel A). We decided to proceed with this solution and indeed four clusters differed in the profile of their visual activity while solving the test (**Figure 2**, panel B).

As illustrated in **Figure 2** (panel B), the first two clusters were characterized by generally low visual focus during the process; the only difference between cluster 1 and 2 was more focus within a certain AOI observed in cluster 1. Thus, participants assigned to the first cluster generally entered into the test quickly (low entry time), briefly scanned all elements, and then tended to focus on selected elements. In the case of people from cluster 2, even more quick and scanning-like functioning was observed. Cluster 3 was composed of participants who focused quite intensively on a certain AOI from the very beginning and proceeded around this specific element. Cluster 4 consisted of people with a more analytical approach – it took them a while to focus on a certain element (relatively long entry time and quick first fixation duration) – then they switched between elements (revisits and many fixations with low dwell time within specific AOIs). In other words, cluster 3 consisted of individuals who seemed to more deliberately compare and combine elements, but their process was more dynamic, while cluster 4 suggested a more analytical approach to solving the test (see **Table 2**).

**Table 2:** Profiles of visual activity while solving the test – analysis of clusters.

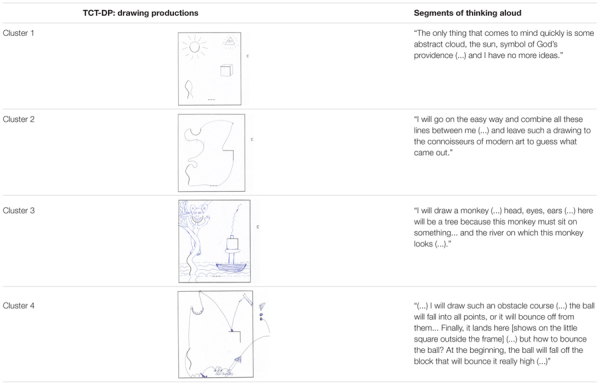

**FIGURE 3:**
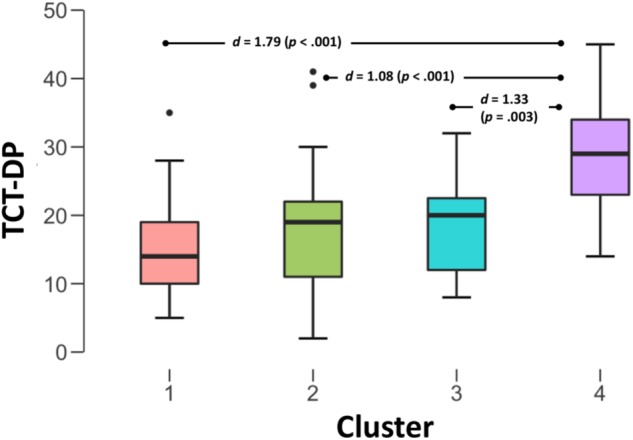
Differences in the total score of the TCT-DP across clusters.

An ANOVA was applied to examine whether the clusters differ in the total TCT-DP score. As illustrated on **Figure 3**, the last, fourth cluster was characterized by not only significantly, but also robustly higher total scores than the three remaining clusters (which did not differ from each other – see **Figure 3**). There was a substantial amount of variance in TCT-DP explained by cluster membership, *F*(3,96) = 10.13, *p* < 0.001, ω^2^ = 0.22, thus indicating that even relatively simple information about visual activity while solving the test is able to robustly predict the scores obtained by test participants.

### Metacognition While Solving the Test

In an effort to examine the more dynamic subprocesses engaged in the creation of the drawing, two coders (first and third authors) independently scored a number of relevant characteristics of this process among a subset of 40 participants – those who obtained the lowest scores in TCT-DP (*n* = 20, *M* = 9.55, *SD* = 1.91) and those with the highest scores (*n* = 20, *M* = 32.30, *SD* = 5.19). Although we classified participants solely based on their total scores, there was a significant and substantial overlap with the clusters described above. In the low-TCT-DP group, none out of 20 participants came from the fourth cluster, while 11 people (55%) were previously assigned to the first cluster. Half of the high TCT-DP group came from the fourth-cluster members (*n* = 10) and this difference in distributions was highly significant, χ^2^(*df* = 3, *N* = 40) = 16.37, *p* < 0.001, Cramer’s Φ = 0.64.

The description of coded categories is provided in **Table 3** together with descriptive statistics and reliabilities. The coders watched short, recorded movie clips and coded participants’ statements accordingly with the number of proposed segments of behaviors, categorized into three larger groups – exploratory activities, decision-making and control activities, and affective-evaluatory activities. Although in general these three groups of meta-regulators were indeed observed in a rather subsequent manner, i.e., in most cases, exploratory activities preceded decision-making / control and affective-evaluatory activities, we note that several exceptions from such a step-by-step pattern were observed. Therefore, even if later on we analyze the differences between groups and categories in a processual manner, we emphasize that the process was not necessarily linear, and the phases should be treated in a much more dynamic and reciprocal way.

**Table 3:** Meta-regulation during the creative process – examples of coded segments of participants thinking aloud statements with reliability and descriptive statistics.

Segment code		Example	Overview	Reliability	*M* (*SD*)
Exploratory activities	Strategic exploration	“I’m not sure what could it be... maybe a flower?”	Talks to oneself before drawing, analyzing the graphic elements placed on the test sheet	α = 0.97	0.56 (0.94)
	Exploration in hand	‘I add lines to the semicircle and now I know that it will be a guitar” [combines the semicircle with the curve line].	Discovers the relationship between graphic elements placed on the test sheet while drawing	α = 0.94	0.93 (2.06)
Decision-making and control activities	Planning	“Now maybe I’ll draw something that will look like helix. This will make it a little more scientific”	Provides justification for planned activity	α = 0.97	1.58 (1.93)
	Correction	[Improves drawing of the sun and says] “maybe it will make the moon and the night.”	Introduces amendments to the proposed solution	α = 0.83	0.45 (0.96)
	Reporting and control	“Could this be a square? [wonders]. Ok, I’ll come back to it later.”	In a controlled way, postpones the execution of some activities related to the task completion	α = 0.95	4.38 (2.77)
	Elaboration	”[Returns to the drawn face] I’ll finish drawing a face and a cap I will do on his head... with a visor.”	Returns to the proposed solution and elaborates it	α = 0.99	1.45 (2.11)
Affective-evaluatory activities	Expressing emotions of a challenge	“I’ll think of something... I can draw whatever I want.’	Treats the test task as a challenge	α = 0.77	0.23 (0.53)
	Expressing emotions of hesitance and uncertainty	“Can it be so simple? I think that it is impossible to do anything about it anymore…”	Seeking approval for proposed solutions	α = 0.94	0.88 (1.20)
	Positive self-evaluation of skills	“The simplest solutions are the best, I cleverly outplayed it.”	Positively evaluates own competencies associated with the performed test task and expresses satisfaction	α = 0.72	0.08 (0.27)
	Negative self-evaluation of skills	”I cannot draw. I don’t have such imagination!”	Negatively evaluates oneself and own competencies needed to solve the test task	α = 0.91	0.30 (0.61)
	Global evaluation	[At the end of solving the test says] “probably it is not too original but... it is simple.”	Evaluates proposed comprehensive test solution	α = 0.73	0.08 (0.27)
	Evaluation of partial solutions	“It does not look like a butterfly... Oh, well! Let’s say it’s a butterfly.”	Evaluates the partial solution while solving the test	α = 0.97	1.50 (1.06)

**FIGURE 4:**
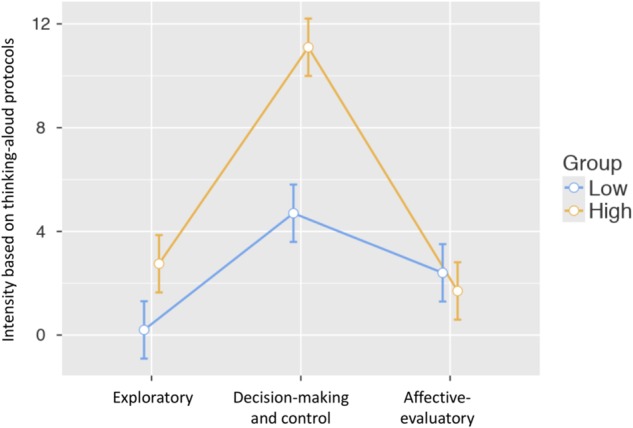
Profiles of metacognitive strategies among low-versus-high scorers in the TCT-DP.

To explore potential differences between participants with the highest and the lowest scores in TCT-DP, a mixed 3 × 2 ANOVA was used. Three groups of meta-cognitive strategies (exploratory, decision-making, affective-evaluatory) served as within-person factors, while the group (low versus high TCT-DP scores) became a between-person factor. There were significant and strong differences between the intensity of different meta-cognitive processes, *F*(2,76) = 97.3, *p* < 0.001, ω^2^ = 0.60. As illustrated on **Figure 4**, there were significantly more expressions that focused on control and decision-making during the process (*M* = 7.90, *SE* = 0.53) than those focused on the exploratorily-generative and evaluatory talk (*M* = 1.48, *SE* = 0.30 and *M* = 2.05, *SE* = 0.32, respectively). We also observed a robust main effect of the group, *F*(1,38) = 27.4, *p* < 0.001, ω^2^ = 0.40, which demonstrated that participants who obtained high scores in TCT-DP were those who outperformed their counterparts with low scores (*M*_low_ = 1.48, *SE*_low_ = 0.30, *M*_high_ = 7.90, *SE*_high_ = 0.53). Finally, there was a significant *Process x Group* interaction, *F*(2,76) = 24.3, *p* < 0.001, ω^2^ = 0.15. Although the profiles looked similar (**Figure 4**), exploratory (*M*_low_ = 0.20, *M*_high_ = 2.75, both *SE*s = 0.42) and decision-making/control activities were much more profound within the group that obtained high scores in TCT-DP (*M*_low_ = 4.70, *M*_high_ = 11.10, *SE*s = 0.75), while the level of affective-evaluatory activities was similar in both groups (*M*_low_ = 2.40, *M*_high_ = 1.70, *SE*s = 0.45). Although we did not observe between-group differences in terms of emotion-based statements during the process, a more detailed analysis showed that there was marginal difference in favor of high-scorers in terms of treating the task as a challenge [Welch’s *t*(*df* = 26.66) = 1.52, *p* = 0.07, one-tailed, Cohen’s *d* = 0.48], and a significantly higher level of uncertainty related statements in the low-TCT-DP group [*t*(*df* = 21.69) = 3.79, *p* < 0.001, Cohen’s *d* = 1.20].

### Meta-Cognitive Strategies and Eye-Tracking During the Creative Process

The last step of our analyses examined the extent to which the observed metacognitive strategies are related to visual activity during the creative process, as measured by eye-tracking scores (see **Table 4**). We used a correlational analysis to examine bivariate relationships and regression analysis to control for the covariance among ET indices. As dwell time was almost perfectly correlated with the number of fixations (*r* = 0.98), we excluded the fixation count from our regression models to avoid multicollinearity.

**Table 4:** Metacognition and eye-tracking – a summary of correlation and regression analyses with intensity of metacognitive strategies regressed onto eye-tracking scores.

	Metacognitive strategies during the process					
	Exploratory		Decision-making and control		Affective-evaluatory	

ET scores	*r*	ß	*R*	ß	*r*	ß
Entry time (ms)	0.23 (–0.09–0.50)	–0.14	0.54^∗∗^ (0.28–0.73)	0.30	0.35^∗^ (0.04–0.59)^b^	0.48^∗^
Dwell time (ms)	0.80^∗∗^ (0.65–0.89)	0.86**	0.59^∗∗^ (0.34–0.76)	0.26	0.03 (–0.29–0.33)	–0.01
First fixation duration (ms)	0.11 (–0.21–0.41)	0.10	0.28 (–0.04–0.54)	0.18	–0.01 (–0.32–0.30)	–0.08
Revisits	0.67^∗∗^ (0.45–0.81)	–0.05	0.57^∗∗^ (0.31–0.75)	0.24	–0.06 (–0.37–0.25)	–0.12
Fixation count	0.76^∗∗^ (0.58–0.87)	a	0.58^∗∗^ (0.32–0.75)	a	0.02 (–0.29–0.33)	a
Dwell time within AOI (%)	–0.20 (–0.48–0.12)	–0.15	–0.36^∗^ (–0.60–0.06)^b^	–0.12	–0.02 (–0.33–0.29)	0.18
Average fixation duration (ms)	0.04 (–0.28–0.35)	–0.04	0.15 (–0.17–0.44)	0.05	0.07 (–0.25–0.37)	–0.01
*R*^2^		0.66		0.55		0.17

*N = 40, a = due to the multicollinearity, fixation count was excluded from the regression model; b = correlation is no longer significant after applying the Holm–Bonferroni correction. ^∗^p < 0.05, ^∗∗^p < 0.001*.

As illustrated in **Table 4**, there were robust, but also diverse correlations between the intensity of metacognitive strategies and ET scores. The level of exploratory behavior during the creative process was excellently predicted by the total dwell time spent on exploring all AOIs (*r* = 0.80, ß = 0.86, *p* < 0.001). Interestingly, although bivariate correlations demonstrated that exploratory statements were also related to a number of revisits (*r* = 0.67, *p* < 0.001), this effect disappeared in regression analysis (ß = -0.05).

A number of statistically significant correlations was observed between decision-making and control activities and ET indices – the intensity of this metacognitive strategy was linked to a later entry time (*r* = 0.54) and longer dwell time overall (*r* = 0.59), the number of revisits (*r* = 0.57) and fixations (*r* = 0.58, all *p*s < 0.001), while being negatively correlated with the percentage of time spent on single element (*r* = -0.36). However, when we controlled for the covariance between ET indices, only the entry time marginally predicted decision-making and control activity (ß = 0.30, *p* = 0.056). Affective-evaluatory behaviors during the process were significantly related only to entry time – the later it was, the higher the affective-evaluatory behaviors were (*r* = 0.35, ß = 0.48).

## Discussion

How does the creative process look when people are struggling with a psychometric creativity test? Is it possible to explore the process using the test of creativity – an instrument routinely used to capture individual differences rather than creative processes? Are thinking-aloud protocols and eye-tracking glasses able to inform our understanding of this process? These three broad questions largely informed our endeavors presented in this exploratory study. Below, we discuss the main findings and their theoretical consequences, with a special focus on promises and risks related to a blended methodology-based analysis of the creative process.

Our results may be summarized with two broad points. First, even very basic scores obtained thanks to the use of eye-tracking methodology were able to explain quite a substantial portion of the variance of the total score in creativity tests. Not only were such parameters as entry time, dwell time, number of revisits between different elements of the tests, and number of fixations on test’s elements, robustly correlated with the total TCT-DP score, but a clear “creative” group emerged when we put eye-tracking scores into hierarchical cluster analysis. This group was characterized by a distinct profile of gaze functioning while solving the test: exploratory on the one hand, but also highly strategic on the other. In short, this cluster combined those who spent a relatively long time while dealing with the test’s material, but also very dynamically switched between its main elements, with many fixations overall, but relatively little time spent on a single element. An illustration provided suggested that individuals who solved the TCT-DP in this way looked for a more complex and interpretable solution rather than simply continuing the drawing. And although this line of reasoning is speculative, it is supported by our subsequent analysis of the meta-regulators during the process.

The second main observation refers to the reports from the thinking-aloud protocols during the process. We categorized them into a wide range of specific categories that described different behaviors and strategies observed across the different phases of the process. More synthetically, however, all these detailed categories were classified into exploratory activities, identifiable especially during the initial phases of the process, decision-making and control activities – the most severe during the whole process, as well as affective-evaluatory activities – visible not only during the final stages, but in fact, dynamically present during the entire process as well. When we compared individuals, who scored the highest and the lowest in the TCT-DP, it became apparent that the differences between groups were primarily related to the first two groups of strategies and activities. High TCT-DP scorers explored the possible ways of solving the test more intensively, but also put much more energy into the continuous assessment whether their initial drawings fit into the goal. Importantly, though, this goal was not always clear in advance. In other words, for many participants who created the most creative drawings it was not necessarily obvious what should be drawn from the very beginning. Therefore, although their activity was goal-directed, the goal was quite general (“to create something interesting”) rather than specific in terms of the actual theme of their drawings. In a sense, initially this process was blind, spontaneous, and chaotic (Simonton, 1999, 2010), but thanks to the executive strategies to control and order it, it became quite analytical and effective (Benedek, 2018). Individuals who scored highly also demonstrated higher challenge-related statements that suggest their higher self-efficacy and engagement (e.g., Beghetto and Karwowski, 2017; Karwowski and Beghetto, 2018).

On a theoretical as well as methodological level, our results may open new avenues of investigation for the creative process. On the one hand, the presented approach may be promising for researchers who are still looking for more dynamic, accurate, and ecologically valid creativity process assessment (Plucker and Renzulli, 1999). On the other, we attempt to conduct a micro-analysis of attentional patterns during a drawing-production process using cluster analysis and demonstrating various patterns of attentional processing of visual information.

Could these findings inform our theorizing about solving creativity tests or creative process in a more general way? We posit that although it is likely more challenging than the traditional psychometric approaches, such a blended methodology holds the potential to enrich our understanding of the dynamics of creative processes in a wide range of spheres – from solving creativity tests, all the way to a more general process of creative learning (see Karwowski, 2018). We do not suggest that conclusions that stem from mixed methods applications are always straightforward or consistent across methods. Quite the opposite, very often they seem a little chaotic and contradictory. Even if this is true, however, in our perception the blended methodology holds the promise of enriching our understanding of the creative process, especially in comparison with static, output-based assessment of creativity tests.

When interpreting the present findings, a number of strengths and limitations should be considered. Among its strengths, we see that by measuring the creativity process in real-time (Schwarz, 2012), we may potentially reduce recall and retrospective biases and thus collect more valid and reliable data. Moreover, we emphasize the potential of dynamizing creativity test for capturing the ongoing and shifting nature of creativity process and its nuances. For instance, such a triangulated and combined approach may be successfully applicable to exploring the interplay between attentional processes and regulatory self-beliefs (i.e., creative metacognition: Kaufman and Beghetto, 2013) during creative thought. Moreover, we suggest that simultaneously tracking eye movements or physiological responses and examining whether it corresponds with patterns of self-regulation or self-learning strategies uses allows us to investigate the creativity process via a more complex and holistic design.

However, our research had certain limitations as well. First, to analyze the creative process we used only one type of creativity measurement, TCT-DP (Urban and Jellen, 1996). Thus, it is possible that the specific structure of this drawing test may evoke a specific profile of gaze distribution or self-regulation strategies. To test the generalizability of our findings beyond this *bottom-up* interpretation, other creativity tests or open tasks should be used in future studies. What is more, although in the presented study the think-aloud method did not influence participant performance, we still keep in mind that, specifically during drawing activity, simultaneous verbalization may interfere with the creative process (Lloyd et al., 1995).

As the present study is exploratory, future research is necessary to incorporate relevant moderating and mediating factors, such as creative self-beliefs (Karwowski and Beghetto, 2018) or experience in drawing. This latter factor was unrelated to the results of the TCT-DP in previous studies (Urban and Jellen, 1996), yet it may be important for metacognition. Indeed, as previous studies demonstrated depending on the expertise people differ on organization (meta-regulation) of the creative process (Kay, 1991).

Despite the fact that the overall scores in the TCT-DP test did not differ from those achieved in previous studies conducted in Poland (Karwowski et al., 2016b), it is important to note the potential influence of the instruction we used. As we encouraged the respondents to be “natural about their reactions,” it could be interpreted in different ways by different participants. Previous studies showed that the type of instruction is related to the level of task performance (e.g., O’Hara and Sternberg, 2001; Chua and Iyengar, 2008), and it is possible that while for some of our participants “natural” meant “to be very creative” for others it might have meant quite the opposite – for example esthetically appealing, logical, etc. Although we consider it unlikely that this instruction influenced our findings heavily, future studies should explore this possibility as well.

## Conclusion

This exploratory investigation examined the possibilities of integrating the psychometric approach to studying creativity with an eye-tracking methodology and thinking-aloud protocols, while studying the creative process. Although primarily methodological, we believe that it also illustrates how such blended approaches may inform more substantial theorizing on the process; theorizing that involves not only cognitive, but also metacognitive aspects of the process.

## Author Contributions

DJ contributed to the conceptualization of the study, coding the data, and approval of the final version of the manuscript. MC drafted the manuscript and approved the final version of the manuscript. IL contributed to coding the data and approval of the final version of the manuscript. MK contributed to the conceptualization of the study, data analyses, drafting the manuscript, and approval of the final version of the manuscript.

## Conflict of Interest Statement

The authors declare that the research was conducted in the absence of any commercial or financial relationships that could be construed as a potential conflict of interest.
